# A Machine Learning-Based Decoder Framework for the Cortical Voltage-Sensitive Dye Responses to Retinal Neuromorphic Microstimulation: A Proof-of-Concept Simulation Study

**DOI:** 10.3390/bioengineering13020231

**Published:** 2026-02-16

**Authors:** Keisuke Yamada, Yuina Terakura, Santa Fukuda, Yuki Hayashida

**Affiliations:** Department of Information Engineering, Graduate School of Engineering, Mie University, Tsu 514-8507, Japan; 424m522@m.mie-u.ac.jp (K.Y.); 425m515@m.mie-u.ac.jp (Y.T.); 423d054@m.mie-u.ac.jp (S.F.)

**Keywords:** intracortical visual prosthesis, physiological experiment, retinal neuromorphic spike, voltage-sensitive dye imaging, machine learning-based decoding

## Abstract

Intracortical microstimulation (ICMS) is a promising approach for visual prostheses. We recently proposed using retinal neuromorphic spike trains derived from visual images as ICMS pulse sequences, and preliminarily recorded cortical voltage-sensitive dye (VSD) responses to such stimulation. To examine whether these cortical responses contain image information, we explore the feasibility of machine-learning–based decoding. However, constructing such a decoder requires large-scale datasets linking visual images, spike trains, and cortical responses, which are not yet experimentally available. Therefore, we generated surrogate data with a Wiener-system model that simulates VSD responses of the visual cortex to ICMS pulse trains. A convolutional neural network trained on these synthetic datasets successfully reconstructed images from the simulated cortical responses. This simulation work serves as a proof-of-concept study, demonstrating the computational feasibility of estimating visual information contained in neuromorphic ICMS-evoked cortical activity and providing a foundation for future physiological validation.

## 1. Introduction

Cortical visual prostheses aim to provide artificial vision for individuals with acquired blindness by bypassing the retina and delivering electrical stimulation to the visual cortex via surface electrodes [1,2,3,4] or intracortical microelectrodes [5,6,7,8]. Conventional intracortical microstimulation (ICMS) typically employs fixed-frequency current-pulse trains lasting several hundred milliseconds to induce consciously perceivable phosphenes [5,8], representing an important step toward establishing a functional prosthesis. At the same time, such stimulation protocols may underexploit the millisecond-scale temporal dynamics of neural circuits in the primary visual cortex (V1).

Using voltage-sensitive dye (VSD) imaging at millisecond resolution [9,10], our previous physiological experiments in mouse brain slices demonstrated that a single ICMS pulse delivered into the layer IV of V1 can trigger temporally synchronized action potentials in a neuron population around the stimulation site [11]. This observation implies that cortical neural activity can be temporally modulated on the order of milliseconds. In this context, we recently proposed using retinal neuromorphic spike timing as a design principle for ICMS [12,13] and preliminarily examined neural voltage responses to such stimuli in V1 of mouse brain slices [14].

However, a central question is whether, and to what extent, image information can be transmitted to cortical activity through these spike-timing-based pulse train stimuli. A possible strategy for answering this question is to apply a machine-learning (ML) framework [15,16,17,18,19] to test whether cortical VSD responses could be decoded into the original images. Crucially, training a decoder requires large datasets comprising matched visual images, retinal spike trains, and cortical responses, which are not yet experimentally available on a sufficiently large scale. To address this limitation, we previously developed a Wiener-system model that can reliably reproduce cortical VSD responses evoked by ICMS with adequate temporal precision [20]. In the present study, we used this model to generate surrogate datasets linking input images, neuromorphic spike sequences, and simulated VSD responses. These synthetic data enabled us to train a convolutional neural network (CNN) to decode images from simulated VSD responses. While physiological experimental data are not used in training and evaluating the decoder, this work serves as a proof-of-concept study demonstrating the computational feasibility of decoding visual information from cortical response patterns in future physiological experiments.

## 2. Methods

### 2.1. Physiological Experiments

All animal care and experimental procedures were approved by the Animal Experiment Committee of Mie University (approval No. 2021-19-Sai1-Hen1), and conducted in conformity with the Guidelines for Proper Conduct of Animal Experiments by the Science Council of Japan. Detailed methodologies followed those described in our previous reports [11,21], and the specific procedures are briefly summarized below.

Coronal brain slices (300 µm thick) containing V1 were prepared from C57BL/6J mice (3–4 weeks old, female, *n* = 8). After staining with an absorption-type voltage-sensitive dye (NK3630), the slices were placed in a recording chamber perfused with oxygenated artificial cerebrospinal fluid for optical recording. Optical VSD signals were recorded from a 154 × 820 µm region covering layers II/III to VI of V1 (see Section 3.1) at a frame rate of 1000 fps, with a spatial resolution of 12 × 64 pixels, using an electron-multiplying charge-coupled device camera (iXon 3 DU-897; Andor Technology, Belfast, UK). For electrical stimulation, a glass micropipette electrode (~6 μm tip diameter, ~1 MΩ) was inserted approximately 150 μm below the cut surface into layer IV of V1, and delivered trains of cathodic-first biphasic current pulses (~1.4 s train duration; 10 µA/phase current amplitude, 0.2 ms/phase pulse duration, no interphase delay). Recordings were averaged over 50–200 repetitions per pulse-train sequence for each slice. In these experiments, the timing sequences of the pulse trains were patterned after the spike-timing sequences previously recorded from mouse off-transient alpha retinal ganglion cells in response to random flickering light [22]. The captured VSD signals were converted into fractional changes in transmittance, denoted as *ΔT*(*tₙ*)/*T*_0_, and subjected to offline analyses. The corresponding VSD images were then averaged over multiple repetitions for each stimulus condition and for no-stimulation trials, and the averaged no-stimulation VSD images were subtracted from those elicited by stimulation.

These processed physiological VSD responses induced by multiple stimulus sequences were used to derive the Wiener-system model [23], as described below (Section 2.2). None of the data from physiological experiments were used for decoding analyses in this study (see Section 2.3).

### 2.2. Wiener-System Model

We employed a Wiener-system modeling approach, which provides a well-established framework for representing nonlinear dynamic systems [23], to simulate cortical VSD responses to ICMS pulses, as reported previously [20]. The Wiener-system model was adopted as a minimal yet sufficiently expressive framework that captures the dominant nonlinear temporal dynamics of cortical VSD responses while allowing robust parameter estimation from limited experimental data. The model consists of a linear dynamic element followed by a static nonlinear element. Mathematically, the model output y(t) is described as:

$$y(t)=f\left\{\left(k*\left(L_{\gamma }*s\right)\right)\left(t\right)\right\}\;,$$

where t denotes time, s(t) is the input pulse-timing sequence, $L_{\gamma }\left(t\right)$ is a temporal Lorentzian function, $k\left(t\right)$ is a temporal linear filter function, f(⋅) is a static nonlinear gain function, and ∗ denotes convolution.

In this model, the temporal Lorentzian function $L_{\gamma }\left(t\right)$, which accounts for temporal broadening of the input impulse, was defined as: $L_{\gamma }\left(t\right)=1/\{1+{\left(t/\gamma \right)}^{2}\}$ where the width parameter γ was set to 0.25 ms [24], corresponding to a full-width at half-maximum of approximately 0.49 ms. The other functions and parameters were experimentally derived from VSD response signals recorded near the stimulation site in the layer IV, based on the simple white-noise analysis [25]. First, the temporal filter function was obtained as the cross-correlation between the Lorentzian-filtered spike train (i.e., $\left(L_{\gamma }*s)(t\right)$) and the measured VSD waveform. Second, the static nonlinear gain function was obtained by fitting the relationship between the linear prediction (i.e., $\left(k*(L_{\gamma }*s)\right)\left(t\right)$) and the measured VSD signal using a modified Softplus function:

$$f\left(x\right)={Softplus}_{\beta }\left\{\frac{p_{1}}{1+e^{-p_{2}(x-p_{3})}}+p_{4}e^{p_{5}x}+p_{6}\right\}.$$

The derivation of these functions and parameters was based on 16 segments of physiological VSD data (each segment ~1.4 s in duration) recorded in response to the mouse retinal spike trains described in Section 2.1. The simulated VSD responses generated by this model were quantitatively compared with corresponding physiological VSD recordings, as described in Section 3.1.

### 2.3. Machine Learning-Based Framework

**Figure 1:**
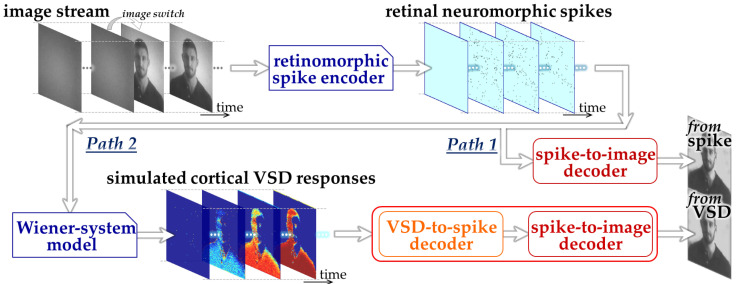
Overall design of the proposed ML-based framework.

We designed an ML-based framework to assess the feasibility of image decoding from the responses simulated by the Wiener-system model, as illustrated in Figure 1. Specifically, in the present study, we constructed two CNN-based decoding pathways: one designed to decode retinal neuromorphic spike images directly into a natural image (Path 1) and the other designed to decode simulated VSD image sequences into a natural image (Path 2). We employed three-dimensional CNN architectures [26] to effectively capture the spatiotemporal dynamics inherent in both neuromorphic spike patterns and VSD signals. Path 1 represents the decoding of visual information prior to cortical transformation and serves as a reference condition based on our previous work [13,22]. Path 2 extends this framework by explicitly incorporating a cortical processing stage via the Wiener-system model, thereby enabling evaluation of how cortical population responses influence decodability.

The CNN in Path 2 was implemented as a two-stage architecture consisting of a front-end block (“VSD-to-spike decoder”) and a back-end block (“spike-to-image decoder”). This hierarchical design reflects the reverse structure of the encoding process, in which cortical responses can be interpreted as transformed representations of retinal spike activity. By decomposing the decoding task into these two stages, each subnetwork is constrained to learn a more specific and interpretable mapping, which facilitates stable training and reduces the risk of overfitting given the limited dataset size.

The procedure of dataset generation and decoder training mainly consisted of three stages. First, natural images were prepared from the royalty-free stock media websites, Pexels, https://www.pexels.com (accessed on 6 February 2023) and Pixabay, https://pixabay.com (accessed on 4 October 2024) and retinal neuromorphic spike patterns in response to these images were generated using the retinomorphic spike encoder developed in our previous studies [13] (upper part in Figure 1). This spike encoder emulates a retinal circuit model adopted from our previously developed retinomorphic hardware system [27], and consists of three main components (1) a delayed difference of two Gaussian spatial filters, (2) biphasic temporal filtering, and (3) spike generation based on the Izhikevich neuron model [28]. The combination of spatial and temporal filtering simulates the generator signals transmitted from retinal bipolar cells to ganglion cells, while the spiking model transforms the resulting continuous-time signals into point-process spike trains with physiologically relevant firing properties [28]. This model architecture allows the parameters to be tuned to reproduce the firing characteristics of mouse off-transient alpha retinal ganglion cells [22]. Second, by inputting the neuromorphic spike patterns to the Wiener-system model (Section 2.2), we simulated cortical VSD responses [20] (lower-left part in Figure 1). Third, using the synthetic datasets linking input natural images, the retinal neuromorphic spike patterns, and the simulated VSD responses, CNNs were trained and evaluated on these datasets to examine whether image information could be decoded from the simulated VSD responses as well as the neuromorphic spikes (lower-right part in Figure 1).

In the first stage, an image stream composed of a full-field gray image followed by a natural image was presented to the retinal spike encoder, and emulated spike trains over 120 × 120 units of a retinal ganglion-cell model [13] were obtained for 150 ms after the image switch. In the present study, spike generation was emulated with a calculation time step of 0.5 ms, and the resulting spike events were combined at 5 ms intervals, yielding a set of “spike images” with 120 × 120 pixels in the spatial domain and 30 frames in the temporal domain. These spike-image data were obtained for 400 different natural images. A representative example of a spike-image sequence is shown in Section 3.1.

In the second stage, since the experimentally derived Winer-system model has the same temporal resolution of 1 ms as in our VSD imaging setup (see Section 2.1 and Section 2.2), the spike-timing sequences were upsampled from 5 ms to 1 ms resolution and then fed as inputs to the Wiener-system model. The simulated cortical responses for 1000 ms were obtained from the model and were down-sampled to 5 ms resolution. This simulation was repeated for each pixel, yielding a set of simulated-response images with 120 × 120 pixels in the spatial domain and 200 frames in the temporal domain. Likewise, these simulated-response images were obtained for 400 different natural images. A representative example of a simulated-response image sequence is shown in Section 3.1.

In the third stage, the above-mentioned 400 matched datasets, each comprising a natural image, the corresponding retinal neuromorphic spike image stream, and the simulated-response image stream, were divided into 320 datasets for training and 80 datasets for testing the CNNs. The front-end and back-end blocks were first trained separately using loss functions based on the binary cross-entropy and the Structural Similarity Index Measure (SSIM) [29], respectively. The two pre-trained blocks were then connected sequentially to form a single CNN for Path 2. Subsequently, the front-end block was re-trained with the parameters of the back-end block fixed, followed by re-training of the back-end block with the parameters of the front-end block fixed, both using the SSIM-based loss function. This two-step training procedure was repeated for three rounds. The Adaptive Moment Estimation (Adam) optimizer [30] was used with a learning rate of 1 × 10^−4^ or 0.5 × 10^−4^ and a batch size of 2, with the number of epochs determined adaptively by early stopping based on monitoring the F1 score or SSIM.

The CNN architectures are summarized in Table 1 and Table 2. The CNNs were implemented in Python 3.10.0 using TensorFlow/Keras 2.10.0 and executed on a Windows 11 system (version 24H2, build 26100) equipped with an Intel^®^ Core™ i7-13700F CPU (2.10 GHz), 32 GB RAM, and an NVIDIA^®^ GeForce RTX 4070 GPU (12 GB VRAM; graphics clock ~2.48 GHz, maximum 3.11 GHz; driver version 572.61). CUDA 11.2 and cuDNN 8.1.1 were used for GPU computation.

## 3. Results

### 3.1. Dataset Synthesis

**Figure 2:**
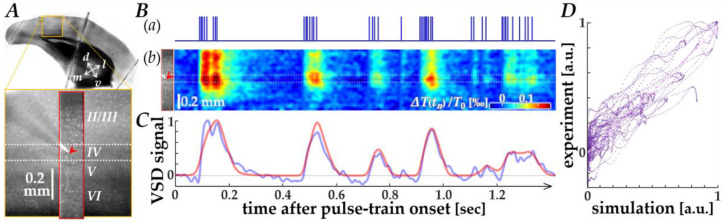
Comparison between physiological and simulated VSD responses. (**A**) Image of the mouse V1 slice (orange square) showing the region of interest for VSD recording (red rectangle). The red arrowhead indicates the position of the microelectrode tip in layer IV. (**B**) Space-time analysis of the responses. (**a**) The input current pulse train patterned after neuromorphic spikes. (**b**) Space-time plot of the experimentally recorded VSD images, showing depolarizing responses (reddish color). The red arrowhead corresponds to the electrode position. (**C**) Time course comparison of the VSD response near the electrode tip. The blue trace represents the experimentally recorded response, and the red trace represents the simulated response generated by the Wiener-system model. (**D**) Scatter plot of experimental versus simulated VSD signal data points. The pink dash-dotted line represents the linear regression fit.

We first verified data correspondence between the cortical VSD responses recorded in physiological experiments and those simulated using the Wiener-system model [20], as illustrated by representative results in Figure 2. In V1 of a mouse brain slice (orange square in panel A), membrane voltage changes in the region of interest (red rectangle) were recorded using VSD imaging, while current pulses patterned after neuromorphic spikes (panel B(a)) were delivered into layer IV through a microelectrode tip (red arrowheads in panels A and B(b)). Figure 2B(b) shows the space-time plot of the recorded VSD images, in which depolarizing responses (reddish color) to the pulses are observed.

In Figure 2C, the time course of VSD responses near the electrode tip (blue trace) is compared with that of the simulated response using the Wiener-system model (red trace), showing a reasonable fit, particularly during the first several hundred milliseconds. A scatter plot of experimental versus simulated VSD signal data points (Figure 2D) showed a clear linear relationship (pink dash-dotted line), yielding a Pearson correlation coefficient of 0.913 (*p* ≈ 0, *n* = 4 traces). The parameters of the Wiener-system model used in this study were derived from sixteen different traces obtained from eight slice samples, with a Pearson correlation coefficient of 0.795 (*p* ≈ 0), indicating that the present model adequately agrees with the physiological response properties.

**Figure 3:**
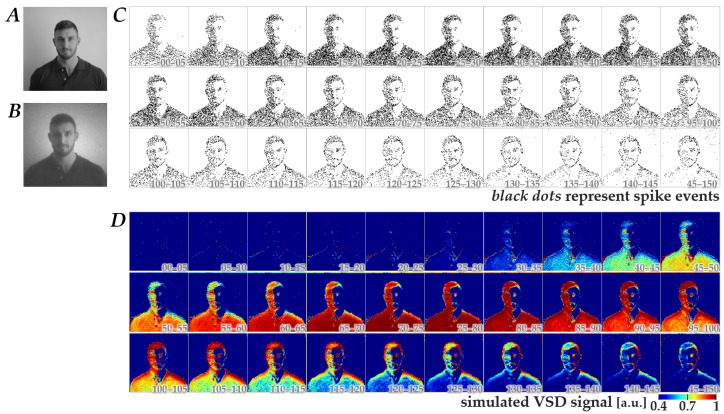
Representative dataset used for CNN training. (**A**) Original natural image obtained from the database. (**B**) Preprocessed natural image derived from the image in panel A by applying a spatial filter based on the photoreceptor-cell-layer model. (**C**) The corresponding retinal neuromorphic spike image stream generated from panel B. Black dots represent spike events. (**D**) The simulated cortical VSD response image stream calculated from panel C using the Wiener-system model. Numbers in each image in panels (**C**,**D**) represent the time (ms) after switching from a full-field gray image to the natural image.

Using the Wiener-system model verified as described above, we next generated simulated-response images in response to spatiotemporal patterns of retinal neuromorphic spikes. Figure 3 shows a representative dataset comprising a natural image (panel B), the corresponding neuromorphic spike image stream (panel C), and the simulated-response image stream (panel D). The image in panel B was obtained from an image in panel A, which was originally in database format (see Methods) and was preprocessed with a Gaussian spatial filter based on the photoreceptor-cell-layer model [13]. As shown in panels C and D, the neuromorphic spikes and simulated-response signals exhibited dynamic changes in spatial patterns, which largely diminished within 150 ms after the onset of natural image display. This tendency was consistent across 400 images, suggesting that the essential information of the images could be encoded within this time window, at least under the stimulation conditions used in the present study.

### 3.2. Decoding Performance

**Figure 4:**
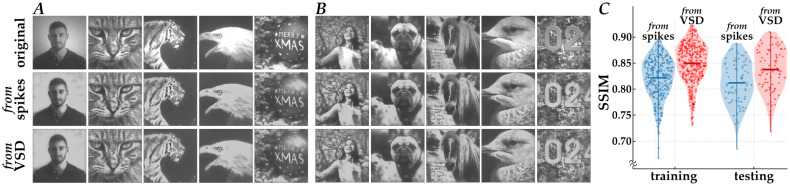
Decoding performances of the CNN models. (**A**,**B**) Representative input natural images (upper row, “original”) and images decoded from the neuromorphic spikes (middle row, “*from* spikes”) and the simulated VSD responses (lower row, “*from* VSD”). (**C**) Violin plots of SSIM distributions for the decoded versus original images.

After training the CNNs using the 400 simulated datasets described above, their decoding performance was examined. Figure 4 shows examples of the original natural images (upper row; “original”), the decoded images from neuromorphic spikes using the CNN in Path 1 (middle row; “*from* spikes”), and the images decoded from simulated VSD signals using the CNN in Path 2 (lower row; “*from* VSD”), for both training datasets (panel A) and testing datasets (panel B). Overall, the spatial patterns of contrast in the decoded images closely resembled those in the original images, although some fine-scale structures and the global contrast level differed. In addition, contrast edges in the original images appeared enhanced in the decoded images, consistent with the spatial band-pass filtering property of retinomorphic image processing [13,27].

Figure 4C summarizes the quantitative evaluation of reconstruction accuracy based on SSIM scores. The CNN in Path 2 (“*from* VSD”) achieved mean SSIM values of 0.85 ± 0.03 for the training dataset (mean ± s. d., *n* = 320) and 0.84 ± 0.03 for the testing dataset (*n* = 80), with a minimal generalization gap (Δ ≈ 0.01) and low deviation, indicating stable reconstruction without overfitting. The CNN in Path 1 (“*from* spikes”) also reconstructed images with reasonable fidelity, yielding mean SSIM values of 0.82 ± 0.04 (training) and 0.81 ± 0.04 (testing).

**Figure 5:**
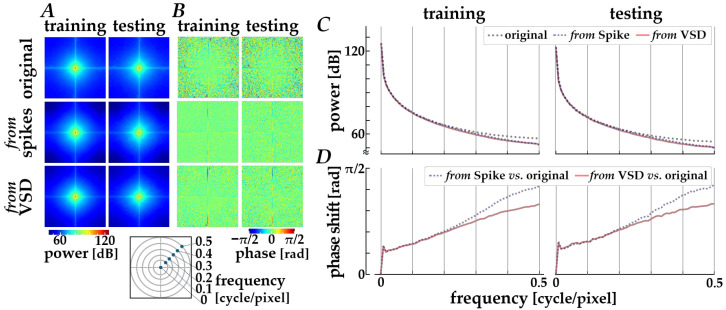
Frequency-domain analysis of decoded images. (**A**) Orientational distributions of spatial power obtained from the two-dimensional Fourier spectra. (**B**) Spatial phase distributions in the frequency domain. In panels (**A**,**B**), columns correspond to the training (left) and testing (right) datasets, and rows show the original images (top), images decoded from spikes using Path 1 (middle), and images decoded from simulated VSD signals using Path 2 (bottom). (**C**) Radially averaged power magnitude plotted as a function of spatial frequency. (**D**) Phase difference between the original and decoded images as a function of spatial frequency. In panels (**C**,**D**), black dotted lines, blue dashed lines, and red solid lines represent the original images, the images decoded from spikes, and those decoded from simulated VSD signals, respectively.

The decoding properties were further assessed in the frequency domain by performing a spatial Fourier transform analysis. The spectral characteristics averaged across the original images (“original”) and across the images decoded via Path 1 (“*from* spikes”) and Path 2 (“*from* VSD”) are compared, as shown in Figure 5. Compared with original images, the decoded images from either spikes or simulated signals are slightly attenuated in the power distributions along diagonal orientations (panel A) and in the radially averaged power magnitude (panel C) in relatively high frequency range (>0.2 cycles/pixel). Also, the phase profiles (panel B) show a loss of fine-grained texture in the spatial phase distributions for the decoded images when compared with those for the original images, implying a loss of phase complexity in the decoded images and a systematic phase shift due to the encoding and decoding processes. The systematic phase delay was verified by plotting the phase difference between the original and the decoded images, as shown in the panel D. As the spatial frequency is higher, the phase difference is increased, especially for the decoded images from spikes.

These results demonstrate that the proposed framework, combining a Wiener-system model with CNN-based decoding, provides a feasible approach to test the feasibility of extracting image information from simulated cortical responses and establishes a basis for future application to physiological VSD data.

## 4. Discussion

In the present study, we trained the CNN to decode the input image from the cortical VSD responses simulated with the experimentally derived Wiener-system model. While physiological experimental data were not used in the decoding analyses study, the results suggested the computational feasibility of decoding visual information from cortical responses. In addition, the proposed decoder framework can be directly applied to experimentally measured VSD data, enabling future physiological validation.

In a recent clinical study on the ICMS-based visual prosthesis [8], stimulation pulses were delivered at a fixed frequency of 300 Hz for a fixed duration of 166 ms, resulting in a fixed number of stimulation pulses (i.e., 300 pulses/s × 166 ms ≈ 50 pulses). Consequently, this fixed stimulation was applied in an all-or-none manner at selected electrode positions to induce phosphene patterns, allowing the patient to detect contrast edges in input images [8].

**Figure 6:**
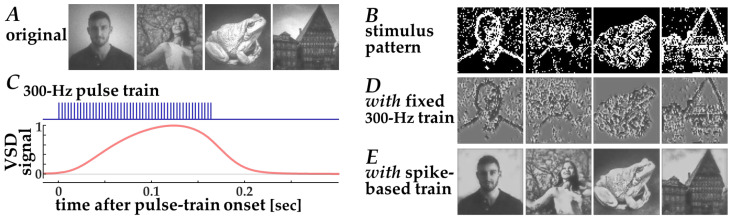
Decoding of images from simulated VSD signals in response to fixed 300-Hz pulse trains based on the standard stimulation strategy [8]. (**A**) Original input images, (**B**) Spatial patterns of the target pixels for stimulation, (**C**) Simulated VSD signals (lower, red trace) in response to the fixed 300-Hz pulse train (upper, blue trace), (**D**,**E**) Images decoded from simulated VSD responses to stimulation with fixed 300-Hz pulse trains (**D**) and with neuromorphic spike-based pulse trains (**E**).

The efficacy of this stimulation strategy could be evaluated using the present simulation framework (Figure 6). First, contrast edges in the input natural images (Figure 6A) were extracted by thresholding after Laplacian filtering. Second, target pixels for stimulation were selected such that the total number of stimulation pulses was matched to that used in the neuromorphic stimulation. Third, the resulting stimulation patterns (Figure 6B) were converted into simulated VSD response signals using the Wiener-system model (Figure 6C). Finally, decoded images were obtained using the CNN decoder (Figure 6D). Because this fixed-frequency stimulation scheme relies on binary stimulation patterns—where stimulation is either present or absent at each electrode location—the resulting VSD response patterns and decoded images exhibit binarized spatial structures. Consequently, fine-grained image details originally present in natural images are less preserved in the reconstructed images (Figure 6D) when compared with those based on the spike-based stimulation (Figure 6E). These results indicate that conventional fixed-frequency stimulation necessarily reduces visual information to binary edge cues, thereby limiting its capacity to transmit more complex visual features.

By contrast, retinal neuromorphic spike-train stimulation has the potential to serve as an effective design principle for ICMS [12,13]. We consider that the retinal neuromorphic spikes, which preserve essential temporal structure inherent in retinal encoding [22,32,33], may be better aligned with the intrinsic dynamics of cortical neurons [34] than conventional fixed stimulation schemes. By embedding image information in the timing of stimulation pulses, such neuromorphic ICMS might be able to effectively drive cortical responses that retain decodable structure at the population level, even though individual neuronal responses are not explicitly resolved. In this context, the decoding results obtained with neuromorphic spike-train stimulation provide important insight into how temporal encoding influences the structure of decoded images. The partial degradation of high-frequency phase structure and the associated phase delay likely reflect limitations of the current encoding and decoding processes, while the preservation of low-frequency phase information appears sufficient to maintain the decodable global image structure. This perspective is consistent with the observation that spatial contrast patterns were reconstructed despite attenuation of fine-scale details. It should be noted, however, that the high SSIM scores of the decoded images (Figure 4) reflect not perceptual fidelity, but rather the topological decodability of the neuromorphic spikes and the surrogate cortical responses.

The effective ~150 ms time window observed in both the simulated neuromorphic spike patterns and the corresponding simulated VSD responses (Figure 3) may reflect temporal integration properties of cortical circuits relevant to visual processing. This finding highlights the importance of examining neuromorphic spike trains derived from different subtypes of retinal ganglion cells, which exhibit diverse temporal response characteristics, as well as the simulated and physiological VSD responses to ICMS pulse trains patterned after those spike trains. Such investigations may clarify how different retinal encoding strategies influence cortical population dynamics and decodability.

The use of VSD imaging in combination with machine learning–based decoding provides access to mesoscopic cortical activity patterns that are difficult to capture using single-unit or multi-unit electrophysiological recordings alone. From this standpoint, the present framework bridges population-level optical measurements and data-driven decoding, offering a complementary approach for evaluating stimulation strategies in cortical visual prostheses. Rather than focusing on individual neuronal responses, this approach emphasizes spatially distributed cortical activity as a substrate for information decoding.

Although the present study focused on visual cortical responses and retinal neuromorphic spike trains, the proposed framework is not limited to the visual modality. In principle, it could be extended to other sensory cortices or alternative neuromorphic encoding schemes, provided that appropriate physiological or surrogate datasets are available. This generality suggests that the framework may serve as a versatile tool for assessing stimulation strategies across a range of neural prosthetic applications.

Despite these advantages, several limitations remain. The present results rely on model-generated surrogate data, and the Wiener-system model necessarily simplifies cortical dynamics by assuming a linear filtering process followed by a static nonlinearity. Consequently, it does not account for complex network-level interactions, recurrent dynamics, or feedback mechanisms. These simplifications may bias decoding performance by favoring stimulus-locked, population-level response components while underrepresenting higher-order cortical dynamics. Furthermore, the Wiener-system model was derived from averaged VSD signals to mitigate optical measurement noise inherent in the VSD imaging technique. While such averaging was essential for accurately identifying system parameters, it may underestimate trial-by-trial neural variability encountered in physiological recordings. Moreover, verification of the proposed decoder framework with physiological VSD responses was not performed. Addressing these limitations will require obtaining additional cortical VSD responses and extensions of the modeling framework to incorporate more complex and realistic cortical dynamics. Taken together, these efforts will enable experimental validation of the proposed decoding framework and support the future development of cortical visual prostheses.

## Figures and Tables

**Table 1:** Architecture of the front-end block of CNN in Path 2.

Layer	Operation [Kernel]	Output	Parameters	Layer	Operation [Kernel]	Output	**Parameters**
0	Input [200 × 120 × 120 × 1]			5	Conv3D [(5 × 3 × 3) × 16]	(200 × 120 × 120) × 16	23,040
1	Conv3D [(7 × 3 × 3) × 16]	(200 × 120 × 120) × 16	1008	6	Batch Norm [*γ*, *β*]	(200 × 120 × 120) × 16	32
2	Batch Norm [*γ*, *β*]	(200 × 120 × 120) × 16	32	7	Conv3D [(3 × 3 × 3) × 32]	(200 × 120 × 120) × 32	13,824
3	Conv3D [(5 × 3 × 3) × 32]	(200 × 120 × 120) × 32	23,040	8	Batch Norm [*γ*, *β*]	(200 × 120 × 120) × 32	64
4	Batch Norm [*γ*, *β*]	(200 × 120 × 120) × 32	64	9	Conv3D [(1 × 1 × 1) × 1]	(200 × 120 × 120) × 1	33

Conv3D: 3-dimensional convolution, Batch Norm: batch normalization [31].

**Table 2:** Architecture of CNN in Path 1 and the back-end block of CNN in Path 2.

Layer	Operation [Kernel]	Output	Parameters	Layer	Operation [Kernel]	Output	Parameters
0	Input [30 × 120 × 120 × 1]			5	Avepooling3D [3 × 1 × 1]	(5 × 120 × 120) × 8	0
1	Conv3D [(30 × 3 × 3) × 4]	(30 × 120 × 120) × 4	1084	6	Conv3D [(5 × 3 × 3) × 16]	(5 × 120 × 120) × 16	5776
2	Conv3D [(30 × 3 × 3) × 4]	(30 × 120 × 120) × 4	4324	7	Avepooling3D [5 × 1 × 1]	(1 × 120 × 120) × 16	0
3	Avepooling3D [2 × 1 × 1]	(15 × 120 × 120) × 4	0	8	Conv3D [(1 × 3 × 3) × 1]	(1 × 120 × 120) × 1	145
4	Conv3D [(15 × 3 × 3) × 8]	(15 × 120 × 120) × 8	4328				

Conv3D: 3-dimensional convolution, Avepooling3D: 3-dimensional average pooling.

## Data Availability

Data is available from the corresponding author upon reasonable request.

## Abbreviations

ICMS	Intracortical microstimulation
V1	Primary visual cortex
VSD	Voltage-sensitive dye
ML	Machine learning
CNN	Convolutional neural network
SSIM	Structural Similarity Index Measure
Adam	Adaptive Moment Estimation
